# Development and Validation of a Rapid Titer Assay for the Oncolytic Virus oHSV2 Expressing a PD-L1/CD3 Bispecific Antibody

**DOI:** 10.3390/v18070694

**Published:** 2026-06-24

**Authors:** Shengjie Zhang, Qingrui Song, Runyang Wang, Rui Chen, Han Hu, Binlei Liu, Yang Wang

**Affiliations:** 1 National “111” Center for Cellular Regulation and Molecular Pharmaceutics, Key Laboratory of Fermentation Engineering (Ministry of Education), Cooperative Innovation Center of Industrial Fermentation (Ministry of Education & Hubei Province), School of Life and Health Sciences, Hubei University of Technology, Wuhan 430068, Chinahuh@hbut.edu.cn (H.H.); 2 Binhui Biopharmaceutical Co., Ltd., Wuhan 430073, China; snlxsbsn@163.com

**Keywords:** oncolytic virus, oHSV2-PD-L1/CD3-BsAb, rapid titer assay, dual-reporter system, NFAT-Fluc, Vero-PD-L1

## Abstract

Oncolytic viruses represent a promising class of anticancer therapeutics, and rapid, accurate quantification of viral titers is critical for ensuring both efficacy and safety during clinical development. Conventional viral titering methods, such as 50% cell culture infectious dose (CCID_50_), are time-consuming and limited in sensitivity, thereby restricting their application in real-time clinical monitoring. This study aimed to develop and validate a rapid titer assay for oHSV2-PD-L1/CD3-BsAb, an oncolytic herpes simplex virus expressing a PD-L1/CD3 bispecific antibody, to support preclinical and clinical monitoring. A dual-reporter cell system was established using Vero-PD-L1-GFP (Vero cells expressing PD-L1 and GFP) cells as target cells and Jurkat-NFAT-Fluc (Jurkat cells expressing NFAT and Fluc) cells as effector cells. Viral infection activates the NFAT signaling pathway, driving Fluc expression, thereby enabling rapid quantification of infectious virus. The assay was evaluated for specificity, limit of detection (LOD), and lower limit of quantification (LLOQ), and compared with the conventional CCID_50_ method. Its applicability was further assessed using clinical simulation samples, including PBMCs and swabs. The rapid titer assay accurately quantified virus at 10^3^ CCID_50_/mL after 8 h of incubation, consistent with CCID_50_ results, while extending the incubation to 18 h improved the LLOQ to 10^2.5^ CCID_50_/mL, demonstrating enhanced sensitivity. The assay exhibited high reproducibility and stability in both PBMC and swab samples, enabling reliable quantification of low-titer virus in complex biological matrices. Compared with CCID_50_, the method substantially reduced assay time (from 3–5 days to 8–18 h) while improving sensitivity and specificity. The developed rapid titer assay for oHSV2-PD-L1/CD3-BsAb provides a sensitive and specific platform for viral quantification. It offers a valuable tool for oncolytic virus development, production quality control, and clinical monitoring, facilitating efficient safety evaluation and risk management in ongoing and future clinical applications.

## 1. Introduction

Malignant tumors remain the leading cause of mortality worldwide, representing a major global health challenge [1]. Oncolytic virus (OV) immunotherapy has emerged as a promising approach that combines selective oncolysis with the activation of systemic anti-tumor immunity. Several OVs have already been approved for clinical use or are under investigation in clinical trials, highlighting their translational potential. Building on this foundation, we previously engineered herpes simplex virus type 2 (HSV-2) as a versatile oncolytic platform. Specifically, we constructed oHSV2 (with ICP34.5 and ICP47 deletions and insertion of human GM-CSF) [2] and oHSV2-PD-L1/CD3-BsAb (with ICP34.5 and ICP47 deletions and insertion of a PD-L1/CD3 bispecific antibody) [3,4]. Both viral constructs have been approved for clinical trials in China and the United States (oHSV2: (NMPA, 2018L02743, FDA, IND 27137; oHSV2-PD-L1/CD3-BsAb: (NMPA 2025LP02059, FDA 28717), enabling investigation across multiple tumor indications.

Precise quantification of viral titer is essential in preclinical and clinical studies to ensure both efficacy and safety of OVs [5]. Conventional titer assays, such as the 50% cell culture infectious dose (CCID_50_), are time-intensive and exhibit limited sensitivity, often leading to inaccurate results for low-titer samples [6,7]. This limitation is particularly pronounced in clinical settings, where samples from injection sites or patient peripheral blood may yield false-negative results [8,9,10]. Furthermore, conventional assays typically require 3–5 days, impeding rapid assessment of clinical samples.

**Figure 1:**
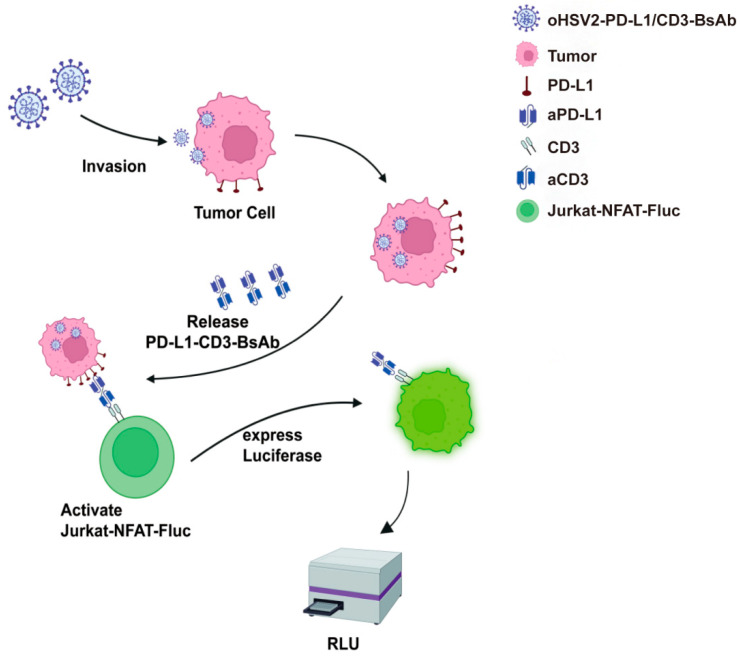
Schematic illustration of the mechanism of the rapid titer assay for oHSV2-PD-L1/CD3-BsAb. This figure was created using the BioRender scientific illustration tool.

oHSV2-PD-L1/CD3-BsAb exerts its anti-tumor activity by expressing a PD-L1/CD3 bispecific antibody that bridges T cells to tumor cells [4]. Leveraging this mechanism, we developed a dual-reporter cell system for rapid viral titer detection. Stable NFAT-expressing Jurkat cells and PD-L1-expressing Vero cells were generated to serve as the detection platform. Infection of PD-L1-positive Vero cells with oHSV2-PD-L1/CD3-BsAb results in viral replication and secretion of the bispecific antibody, which simultaneously engages PD-L1 [11] on Vero cells and CD3 [12] on Jurkat cells, thereby activating NFAT signaling and downstream firefly luciferase (Fluc) expression [13,14]. Quantification of NFAT-induced Fluc activity allowed us to construct a standard curve correlating relative luminescence units (RLU) with the logarithm of viral titer, establishing a rapid and quantitative assay for oHSV2-PD-L1/CD3-BsAb (Figure 1). Validation demonstrated that this method offers high specificity, sensitivity, and reproducibility, and is suitable for rapid viral quantification in clinical simulation samples, including swabs and PBMCs.

This study aims to establish a novel, rapid, and reliable viral titer assay to overcome the limitations of the conventional CCID_50_ method, including its lengthy turnaround time, limited sensitivity, and inaccurate quantification of low-titer samples. This study presents a novel, rapid, and reliable viral titer assay that addresses the limitations of conventional methods and provides a robust tool to support the preclinical and clinical development of bispecific antibody-expressing oncolytic HSV-2.

## 2. Materials and Methods

### 2.1. Ethics Statement

Peripheral blood samples from healthy donors were collected under a protocol approved by the Ethics Committee of Hubei Cancer Hospital (Approval No. 2018-059-007). Written informed consent was obtained from all donors prior to sample collection. The study was conducted in accordance with the Declaration of Helsinki and relevant institutional guidelines.

### 2.2. Cell Lines and Plasmids

A375 (melanoma), A549 (lung adenocarcinoma), ICP4-associated permissive cells, Vero (African green monkey kidney epithelial cells), and Jurkat cell lines were obtained from the Cell Bank of the Chinese Academy of Sciences. Vero cells were used as the primary permissive platform for oncolytic HSV-based virus propagation and functional expression analysis due to their high susceptibility to HSV infection. Human tumor cell lines, including A375 and A549, were employed to evaluate viral expression and oncolytic activity in clinically relevant cancer models, thereby confirming translatability across distinct tumor backgrounds. ICP4-associated permissive cells were utilized to support and assess HSV replication competence under controlled conditions. In parallel, Jurkat T lymphocyte cells were engineered to stably express an NFAT-driven luciferase reporter (Jurkat-NFAT-Fluc) and served as a functional effector system to quantify CD3-mediated T-cell activation induced by the PD-L1/CD3 bispecific antibody bridge. DMEM, RPMI-1640 medium, and fetal bovine serum (FBS) were purchased from Gibco (Thermo Fisher Scientific, Waltham, MA, USA). Plasmids pPBDP-PD-L1-GFP and pPBDP-NFAT-Fluc-CD19 were constructed and maintained in our laboratory.

Supernatants from oHSV2-PD-L1/CD3-BsAb-infected Vero and A375 cells were collected over a 0–48 h time course, and the expression levels of PD-L1/CD3-BsAb in the supernatants at different time points were quantified using ELISA.

Cell cytotoxicity was assessed using the MTT colorimetric assay. A375, A549, ICP4-associated permissive cells, and Vero cells were infected with serial dilutions of oHSV2-PD-L1/CD3-BsAb at an MOI ranging from 0 to 5, and the IC_50_ values of each cell line were compared.

### 2.3. Generation of Stable Reporter Cell Lines

Vero-PD-L1-GFP cells: Vero cells were transfected with pPBDP-PD-L1-GFP using Lipofectamine 3000 (Invitrogen, Invitrogen, Thermo Fisher Scientific, Waltham, MA, USA). Stable integrant were selected with puromycin (1.5 μg/mL, minimum lethal concentration determined empirically). Clonal populations were obtained via limiting dilution. PD-L1 expression was confirmed by flow cytometry using APC-conjugated anti-human CD274 antibody (BioLegend, San Diego, CA, USA), yielding a stable Vero-PD-L1-GFP cell line. Jurkat-NFAT-Fluc cells: Jurkat cells were transfected with pPBDP-NFAT-Fluc-CD19 using Lipofectamine 3000. Stable clones were selected with puromycin (0.8 μg/mL). Clonal populations were established by limiting dilution, and reporter-positive clones were identified by flow cytometry, generating the Jurkat-NFAT-Fluc effector cell line.

### 2.4. Production and Purification of oHSV2-Based Oncolytic Viruses

oHSV2-PD-L1/CD3-BsAb [4] and the oHSV2 [2] virus were constructed and maintained in our laboratory. Viral stocks were propagated in Vero cells at a multiplicity of infection (MOI) of 0.05 for 48–72 h. Culture supernatants were harvested, clarified, and concentrated by high-speed centrifugation for 120 min. Viral pellets were resuspended in IS buffer, titrated, and stored at −80 °C until further use.

### 2.5. Development of Rapid Viral Titer Assay

Vero-PD-L1-GFP cells were seeded in 96-well plates (2 × 10^4^ cells/well) and cultured overnight. Jurkat-NFAT-Fluc cells were added at an effector-to-target ratio of 1:2. Serial dilutions of virus (50 μL/well) were applied, followed by incubation for 8 h or 18 h. Luciferase activity was quantified using a luminescence reader. Dose–response relationships were modeled using a four-parameter logistic equation:

(1) $$y=\frac{A-D}{1+{\left(X/C\right)}^{B}}+D$$

where *X* represents viral titer (log scale) and y represents relative luminescence units (RLU). *A* [15] represents the upper asymptote (maximum response), *B* represents the steepness of the curve at the inflection point, *C* denotes the inflection point (EC_50_), corresponding to the concentration at which 50% of the maximal response is achieved, *D* represents the lower asymptote (baseline response).

### 2.6. Quantitative Real-Time PCR (qPCR) Analysis of oHSV2-PD-L1/CD3-BsAb

The copy number of oHSV2-PD-L1/CD3-BsAb was quantified by quantitative real-time PCR (qPCR). The thermal cycling conditions were as follows: pre-denaturation at 95 °C for 1 min (1 cycle), followed by 30 amplification cycles of denaturation at 95 °C for 15 s and annealing/extension at 60 °C for 30 s. The primer sequences were as follows: F: TGCAGAGCCAGTTCAAGTGTAAG; R: AAGCCACTTTGGATGTGTCATAAA. The probe sequence was FAM–TCAGGCACCTCCCCCAAAAGATGG.

### 2.7. Assay Validation

Specificity: oHSV2-PD-L1/CD3-BsAb and oHSV2 (hGM-CSF) were tested at matched titers under identical conditions. RLU signals were measured after 8 h and 18 h to assess assay specificity. A series of blocking assays and control assays were also performed to verify the specificity of this method: Anti–PD-L1 blocking assay: Vero-PD-L1-GFP target cells were pre-incubated with a neutralizing anti-human PD-L1 antibody to occupy PD-L1 binding sites. Anti-CD3 blocking assay: Jurkat-NFAT-Fluc effector cells were pre-incubated with a neutralizing anti-human CD3 antibody to block CD3 binding sites. NFAT pathway inhibition: Cyclosporin A, a calcineurin inhibitor, was added to the co-culture system to block NFAT dephosphorylation and nuclear translocation. oHSV2 control: Co-culture systems were infected with control oHSV2 lacking PD-L1/CD3-BsAb expression. No-virus control: In co-cultures of Vero-PD-L1-GFP and Jurkat-NFAT-Fluc cells without viral infection (PBS control) and additional negative cell controls: Vero + Jurkat-NFAT-Fluc (PD-L1-negative target control) and Vero-PD-L1 + Jurkat (NFAT-Fluc-negative effector control).

Limit of detection (LOD): Serial dilutions of oHSV2-PD-L1/CD3-BsAb were tested in co-culture systems. LOD was defined as the lowest concentration significantly different from blank controls. Lower limit of quantification (LLOQ): LLOQ was determined using six independent replicates per dilution. Precision (RSD %) and accuracy were evaluated according to standard bioanalytical criteria: Precision (RSD%) is <15% [16], and accuracy ranges from 75% to 125% [17].

### 2.8. Application in Clinical Simulation Matrices

Swab samples: For simulated swab sample collection, sterile swabs were used to apply oHSV2-PD-L1/CD3-BsAb at defined titers (10^5^–10^7^ CCID_50_/mL) onto a gloved index fingertip surface in a controlled manner to mimic clinical skin contamination. Subsequently, a fresh PBS-prewetted sterile swab was used to sample each virus concentration by standardized wiping over the same defined surface area. Then swab samples were analyzed using the developed assay to evaluate matrix performance, LOD, and LLOQ.

PBMC samples: Fresh peripheral blood was collected from healthy donors using EDTA anticoagulation tubes, and peripheral blood mononuclear cells (PBMCs) were isolated by density gradient centrifugation. PBMCs were prepared as pooled samples derived from multiple healthy donors. Cells were infected with serial viral dilutions and cultured for 72 h. Supernatants were collected for viral quantification.

### 2.9. Statistical Analysis

Data are presented as mean ± SD as indicated. Comparisons of more than two groups were performed using one-way ANOVA with Tukey’s post hoc test A *p*-value < 0.05 was considered statistically significant. All experiments were performed with at least three independent biological replicates (n = 3); Each biological replicate included three technical replicates per condition, depending on the assay format (titration curves, swab, and PBMC experiments). Four-parameter logistic (4PL) curves were generated using GraphPad Prism 10, with 95% confidence intervals.

## 3. Results

### 3.1. Cell Line Construction for Assay Development

**Figure 2:**
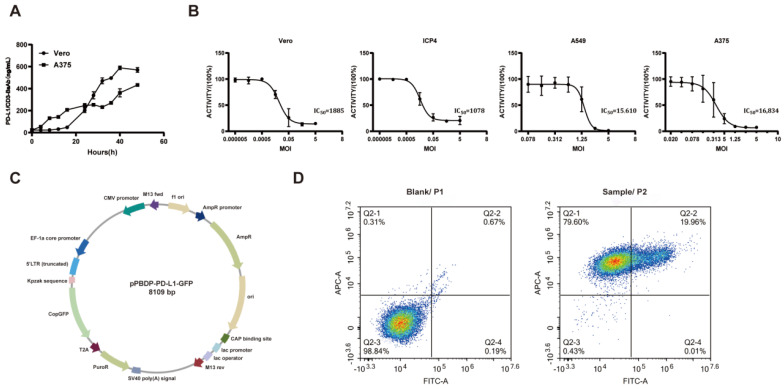
**Construction of the target cell line Vero-PD-L1-GFP.** (**A**) Expression levels of PD-L1/CD3-BsAb in A375 and Vero cells following infection with oHSV2-PD-L1/CD3-BsAb (n = 3, data presented as mean ± SD). (**B**) Cytotoxic activity of oHSV2-PD-L1/CD3-BsAb in different cell lines assessed by MTT assay (n = 5, data presented as mean ± SD). (**C**) Plasmid map of pPBDP-PD-L1-GFP. (**D**) Flow cytometry analysis of PD-L1 positivity in monoclonal Vero cells stably expressing PD-L1 (left: negative control).

**Figure 3:**
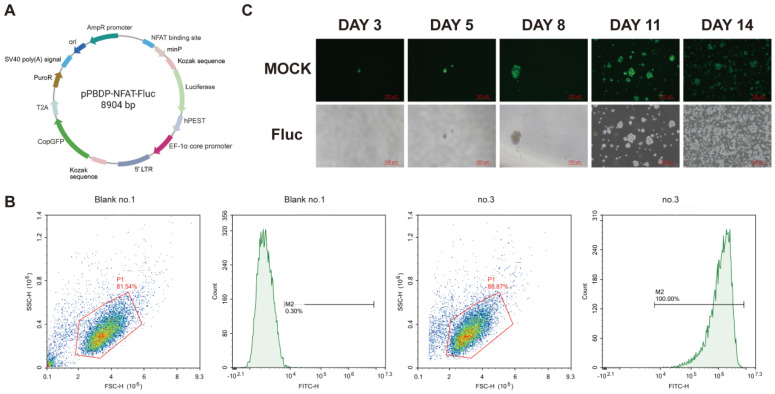
**Construction of the effector cell line Jurkat-NFAT-Fluc.** (**A**) Schematic map of the pPBDP-NFAT-Fluc-CD19 plasmid. (**B**) GFP expression in selected monoclonal Jurkat-NFAT-Fluc cells. (**C**) Flow cytometry analysis of the positivity rate of Jurkat-NFAT-Fluc cells. Cells were first displayed in a dot plot with forward scatter height (FSC-H) on the x-axis and side scatter height (SSC-H) on the y-axis. The target cell population was gated using region P1 to exclude non-target events such as cell debris and aggregates. The P1-gated population was then further analyzed for FITC fluorescence intensity using a histogram (x-axis: FITC-H, y-axis: cell count). A threshold was defined using gate M2 to distinguish FITC-negative and FITC-positive cell populations. Scale bar = 120 μm.

To evaluate PD-L1/CD3-BsAb expression, oHSV2-PD-L1/CD3-BsAb was used to infect Vero and A375 cells, and supernatants were collected at multiple time points. ELISA analysis confirmed that PD-L1/CD3-BsAb was detectable in both tumor cells (A375) and normal cells (Vero), demonstrating efficient viral expression across cell types (Figure 2A). The oncolytic activity of oHSV2-PD-L1/CD3-BsAb was further assessed using the MTT assay in Vero, ICP4-associated permissive cells, A549, and A375 cells. Vero and ICP4-associated permissive cells exhibited lower IC_50_ values, indicating higher susceptibility to virus-mediated cytotoxicity (Figure 2B). Based on these results, Vero cells were selected for stable PD-L1 expression. Vero cells were first transfected with a PD-L1-expression plasmid (Figure 2C), followed by puromycin selection to establish stable PD-L1-expressing monoclonal populations. Flow cytometry analysis confirmed a PD-L1 positivity rate of 99.56%, establishing a robust Vero-PD-L1-GFP cell line suitable for downstream functional assays (Figure 2D). To establish a reporter system for T-cell activation, Jurkat cells were transfected with the pPBDP-NFAT-Fluc-CD19 plasmid, followed by puromycin selection to isolate clonal populations. Flow cytometry analysis identified clone #3 as having the highest NFAT-Fluc positivity, reaching 100% (Figure 3). This clone was used to generate a stable NFAT-Fluc-expressing Jurkat cell line (Jurkat-NFAT-Fluc).

### 3.2. Development of a Rapid Titer Assay for oHSV2-PD-L1/CD3-BsAb

**Figure 4:**
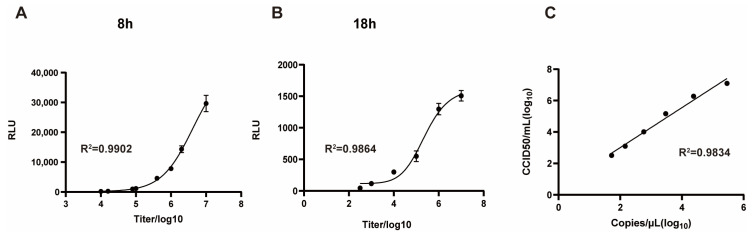
**Generation of the standard curve for the rapid titer assay of oHSV2-PD-L1/CD3-BsAb.** (**A**) Correlation curve between logarithmic viral titer and luminescence intensity after 8 h incubation (n = 3, data presented as mean ± SD). (**B**) Correlation curve between logarithmic viral titer and luminescence intensity after 18 h incubation (n = 3, data presented as mean ± SD). (**C**) qPCR analysis of the linear correlation between viral copy numbers and viral titers measured by the newly established rapid titer assay.

To establish a rapid and quantitative viral titer assay, Jurkat-NFAT-Fluc cells were employed as effector cells, and Vero-PD-L1-GFP cells served as target cells. The cells were co-cultured at an effector-to-target ratio of 1:2 and exposed to serial dilutions of oHSV2-PD-L1/CD3-BsAb. Fluc luminescence was measured at 8 and 18 h post-infection. Analysis revealed a strong correlation between luminescence intensity and the logarithm of viral titer, which fit a four-parameter logistic model: (Equation (1)) with correlation coefficients of R^2^ = 0.9902 and 0.9864, exceeding the predefined threshold of R^2^ > 0.96 (Figure 4A,B). We used the established rapid assay to determine viral titers ranging from 10^2.5^ to 10^7^ CCID_50_/mL. In parallel, qPCR was performed to quantify viral copy numbers at the corresponding titers. Linear regression analysis was then conducted between the viral titers measured by the rapid assay and the qPCR-derived viral copy numbers across different concentrations, demonstrating a strong linear correlation between the two measurements (R^2^ = 0.98) (Figure 4C).

### 3.3. Validation of the Rapid Titer Assay for oHSV2-PD-L1/CD3-BsAb

**Figure 5:**
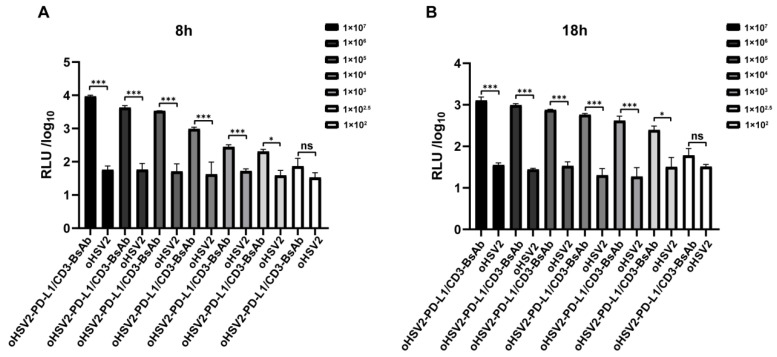
**Specificity validation of the rapid titer assay for oHSV2-PD-L1/CD3-BsAb.** (**A**) Specificity assessment of the assay following 8 h incubation (one-way ANOVA with Tukey’s multiple comparisons test; *** *p* < 0.001, * *p* < 0.05, ns: not significant) (n = 3, data presented as mean ± SD). (**B**) Specificity assessment following 18 h incubation (one-way ANOVA with Tukey’s multiple comparisons test; *** *p* < 0.001, * *p* < 0.05, ns: not significant) (n = 3, data presented as mean ± SD).

**Figure 6:**
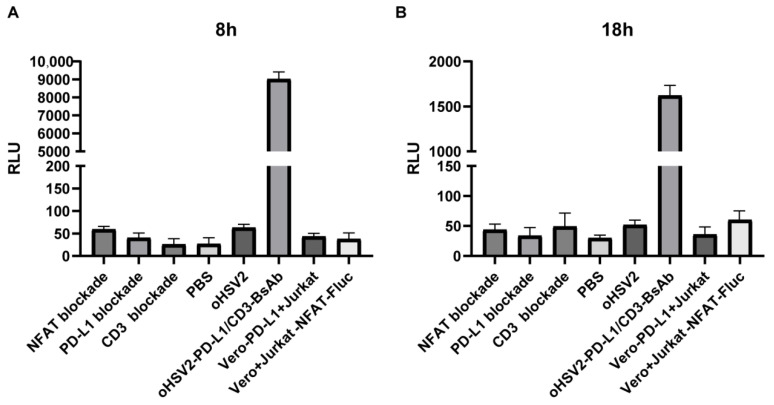
**Effects of different blocking agents, pathway inhibitors, and control treatments on the fluorescence intensity of the newly established assay.** (**A**) shows results after 8 h of incubation, and (**B**) shows results after 18 h of incubation.

**Figure 7:**
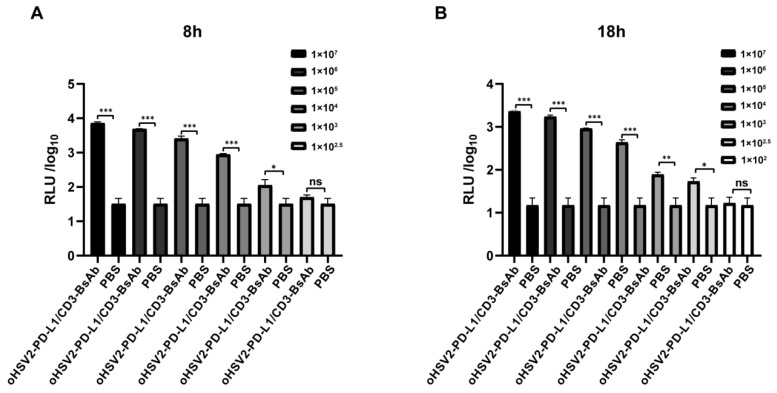
**Limit of detection (LOD) validation of the rapid titer assay for oHSV2-PD-L1/CD3-BsAb.** (**A**) LOD assessment following 8 h incubation (one-way ANOVA with Tukey’s multiple comparisons test; *** *p* < 0.001, * *p* < 0.05, ns: not significant) (n = 3, data presented as mean ± SD). (**B**) LOD assessment following 18 h incubation (one-way ANOVA with Tukey’s multiple comparisons test; *** *p* < 0.001, ** *p* < 0.01, * *p* < 0.05, ns: not significant) (n = 3, data presented as mean ± SD).

For specificity assessment, Jurkat-NFAT-Fluc effector cells were co-cultured with Vero-PD-L1-GFP target cells at an effector-to-target ratio of 1:2 and infected with oHSV2 (expressing hGM-CSF) or oHSV2-PD-L1/CD3-BsAb (expressing PD-L1/CD3-BsAb), followed by serial viral dilutions where applicable. Fluc luminescence was measured after 8 and 18 h of incubation. The assay reliably distinguished oHSV2-PD-L1/CD3-BsAb from oHSV2 at titers above 10^2.5^ CCID_50_/mL, demonstrating high specificity. (Figure 5). Blocking assays, pathway inhibition, and negative control experiments demonstrated that the luminescence signal is specifically generated through PD-L1/CD3 engagement between target and effector cells and is strictly dependent on CD3- and PD-L1-mediated interaction as well as NFAT-driven transcriptional activation, with minimal contribution from non-specific or background activation (Figure 6). For sensitivity evaluation, the limit of detection (LOD) for oHSV2-PD-L1/CD3-BsAb was determined to be 10^3^ CCID_50_/mL at 8 h, improving to 10^2.5^ CCID_50_/mL at 18 h, indicating enhanced sensitivity with prolonged incubation (Figure 7).

The lower limit of quantification (LLOQ) of the newly developed rapid viral titer assay was assessed and benchmarked against the conventional CCID_50_ method. The assay demonstrated an LLOQ of 10^3^ CCID_50_/mL following 8 h of incubation, which improved to 10^2.5^ CCID_50_/mL after 18 h, reflecting enhanced sensitivity over time. In comparison, the CCID_50_ assay exhibited both a detection limit and LLOQ of 10^3^ CCID_50_/mL (Table 1).

### 3.4. Application in Clinical Simulation Samples

To evaluate the assay’s practical applicability, clinical simulation samples were prepared by spiking swabs and PBMCs with oHSV2-PD-L1/CD3-BsAb at titers ranging from 10^3.5^ to 10^7^ CCID_50_/mL. Six consecutive measurements were performed to assess assay performance. For swab samples, the LOD and LLOQ were 10^5^ CCID_50_/mL and 10^5.5^ CCID_50_/mL at 8 h, and 10^4.5^ CCID_50_/mL and 10^5^ CCID50/mL at 18 h (Table 2). In PBMC samples, both 8 h and 18 h incubations yielded an LOD of 10^3.5^ CCID_50_/mL and an LLOQ of 10^4^ CCID_50_/mL (Table 3). These results indicate that the assay is capable of rapid and reliable viral quantification in complex biological matrices, supporting its utility in preclinical and clinical monitoring.

## 4. Discussion

Rapid quantification of oncolytic virus titers is a critical component for ensuring both therapeutic efficacy [18] and safety. During clinical trials, monitoring the presence of oncolytic virus in bodily fluids (e.g., blood), excreta (e.g., urine, feces), and swabs from injection sites [19,20] is essential for evaluating viral shedding and potential transmission risks [5,21]. While quantitative PCR is commonly used to detect viral nucleic acid copies, it cannot distinguish between infectious and non-infectious virus [22]. Therefore, rapid and sensitive measurement of infectious viral titers is necessary for accurate clinical monitoring. Conventional titering methods, such as CCID_50_, are time-consuming (typically requiring 3–5 days), operationally complex, and limited in sensitivity, making them insufficient for real-time clinical surveillance [23].

In this study, we developed a rapid titer assay for oHSV2-PD-L1/CD3-BsAb based on a dual-reporter cell system: Vero-PD-L1 cells as target cells and Jurkat-NFAT-Fluc cells as effector cells. Viral infection activates the NFAT signaling pathway, driving Fluc expression, thereby enabling rapid quantification of infectious virus. Under an 8 h co-culture condition, the assay’s lower limit of quantification (LLOQ) was comparable to that of the conventional CCID_50_ method, accurately detecting 10^3^ CCID_50_/mL. Extending the incubation to 18 h further improved sensitivity, with an LLOQ of 10^2.5^ CCID_50_/mL, demonstrating superior sensitivity relative to CCID_50_. In addition, the assay significantly reduces the detection time, requiring only 8–18 h compared to 3–5 days for conventional methods, thereby improving efficiency and reducing operational costs. It should be noted that the rapid titer assay established in this study relies on the ‘bridging’ mechanism of oHSV2-PD-L1/CD3-BsAb. Following viral infection of target cells, the secreted bispecific antibody (PD-L1/CD3-BsAb) bridges PD-L1 on the surface of target cells and CD3 on effector T cells, thereby activating the NFAT signaling pathway and inducing luciferase reporter gene expression. This indicates that the assay is not directly applicable to other HSV strains or other bispecific antibodies unless the cellular and molecular components are specifically re-engineered.

Validation in clinical simulation samples showed that the assay maintained high stability and reproducibility in both PBMC and swab samples, reliably quantifying low-titer virus in complex biological matrices. Moreover, it allows rapid determination of whether nucleic acid-positive samples contain infectious virus, providing a robust tool for safety monitoring in clinical trials. Compared with conventional titering methods, this rapid assay offers clear advantages in sensitivity, specificity, and turnaround time, supporting oncolytic virus development, manufacturing quality control, and real-time clinical sample monitoring. In the testing of clinically simulated samples, discrepancies were observed between the viral inoculation titers and the measured infectious titers. This may be attributed to differences in matrix recovery efficiency and the accessibility of infectious virions. In the swab matrix, a proportion of viral particles may adsorb to the swab fibers and cannot be fully released during the elution process, ultimately leading to an underestimation of the measured infectious titer. In the peripheral blood mononuclear cell (PBMC) matrix, interactions between viral particles and cellular components may result in partial inactivation or transient retention of the virus, thereby reducing the number of detectable infectious virions.

As oncolytic virus therapies continues to expand across a broad range of solid tumors and hematologic malignancies [24], rapid viral titer assays are expected to become essential tools for regulatory compliance and clinical safety assessment. The highly sensitive, rapid, and scalable detection system established in this study provides a practical platform to support clinical development, dose optimization, and management of potential viral shedding risks.

## Figures and Tables

**Table 1:** Comparison of precision and accuracy between the established rapid titer assay for oHSV2-PD-L1/CD3-BsAb and the CCID_50_ method.

Method	Incubation	Theoretical Titer (log_10_ CCID_50_/mL)	6	5	4	3	2.5
**CCID_50_**	72 h	Precision (RSD %)	1.5	1.3	12	13	-
		Accuracy (%)	108–113	110–114	90–117	90–103	-
**Rapid titer assay**	8 h	Theoretical titer (log_10_)	6.25	5	3.75	3	2.5
		Precision (RSD %)	2.7	3.9	1.8	8.5	-
		Accuracy (%)	100–104	98–108	90–95	79–99	-
	18 h	Theoretical titer (log_10_)	6	4.75	3.5	2.5	2
		Precision (RSD %)	2.7	3.9	3.2	6.2	-
		Accuracy (%)	105–106	102–111	107–112	111–117	-

Note: The lower limit of quantification (LLOQ) was defined as having a precision (RSD %) < 15% and an accuracy within 75–125%. “-” indicates that the viral titer is below the detection limit of the assay; therefore, RSD% and accuracy cannot be calculated.

**Table 2:** Application of the rapid titer assay in swab samples.

Incubation	Inoculated Titer (log_10_ CCID_50_/mL)	7	6.5	5.5	5
**8 h**	Measured titer (log_10_ CCID_50_/mL)	5.24–5.72	5.16–5.53	4.70–5.82	3.03–4.33
	Theoretical titer (log_10_)	6.48	5.98	4.98	4.48
	Precision (RSD %)	3.73	2.84	9.73	16.02
	Accuracy (%)	113–124	108–116	86–106	103–147
**Incubation**	Inoculated titer (log_10_ CCID_50_/mL)	6	5.5	5	4.5
**18 h**	Measured titer (log_10_ CCID_50_/mL)	5.42–4.45	4.29–5.24	3.70–4.62	3.1–3.71
	Theoretical titer (log_10_)	5.48	4.98	4.48	3.98
	Precision (RSD %)	7.74	8.04	9.01	7.31
	Accuracy (%)	101–123	95–116	97–121	107–128

Note: The lower limit of quantification (LLOQ) was defined as a precision (RSD %) < 15% and accuracy within 75–125%.

**Table 3:** Application of the rapid titer assay in PBMC samples.

Incubation	Inoculated Titer (log_10_ CCID_50_/mL)	7	6	5	4	3.5
**8 h**	Measured titer (log_10_ CCID_50_/mL)	5.52–6.65	5.67–6.03	3.98–5.05	3.50–4.18	2.72–3.97
	Theoretical titer (log_10_)	6.85	5.85	4.85	3.85	3.35
	Precision (RSD %)	7.43	2.99	8.65	7.52	16.63
	Accuracy (%)	103–124	97–103	96–122	92–101	84–124
**Incubation**	Inoculated titer (log_10_ CCID_50_/mL)	7	6	5	4	3.5
**18 h**	Measured titer (log_10_ CCID_50_/mL)	6.34–7.61	5.27–5.91	4.53–5.05	3.26–4.01	2.49–3.52
	Theoretical titer (log_10_)	6.85	5.85	4.85	3.85	3.35
	Precision (RSD %)	8.75	4.58	3.74	9.57	14.95
	Accuracy (%)	90–108	99–111	96–107	96–118	95–137

Note: The lower limit of quantification (LLOQ) was defined as a precision (RSD %) < 15% and accuracy within 75–125%.

## Data Availability

Data will be made available on request.
